# The Utility of Cardiac Reserve for the Early Detection of Cancer Treatment-Related Cardiac Dysfunction: A Comprehensive Overview

**DOI:** 10.3389/fcvm.2020.00032

**Published:** 2020-03-10

**Authors:** Stephen Foulkes, Guido Claessen, Erin J. Howden, Robin M. Daly, Steve F. Fraser, Andre La Gerche

**Affiliations:** ^1^School of Exercise and Nutrition Sciences, Institute of Physical Activity and Nutrition, Deakin University, Geelong, VIC, Australia; ^2^Department of Sports Cardiology, Baker Heart and Diabetes Institute, Melbourne, VIC, Australia; ^3^Department of Cardiovascular Medicine, University Hospitals Leuven, Leuven, Belgium; ^4^Cardiology Department, St. Vincent's Hospital Melbourne, Melbourne, VIC, Australia

**Keywords:** cardiotoxicity, cardio-oncology, cardiac imaging, cardiac reserve, exercise echocardiography, exercise ventriculography, exercise CMR

## Abstract

With progressive advancements in cancer detection and treatment, cancer-specific survival has improved dramatically over the past decades. Consequently, long-term health outcomes are increasingly defined by comorbidities such as cardiovascular disease. Importantly, a number of well-established and emerging cancer treatments have been associated with varying degrees of cardiovascular injury that may not emerge until years following the completion of cancer treatment. Of particular concern is the development of cancer treatment related cardiac dysfunction (CTRCD) which is associated with an increased risk of heart failure and high risk of morbidity and mortality. Early detection of CTRCD appears critical for preventing long-term cardiovascular morbidity in cancer survivors. However, current clinical standards for the identification of CTRCD rely on assessments of cardiac function in the resting state. This provides incomplete information about the heart's reserve capacity and may reduce the sensitivity for detecting sub-clinical myocardial injury. Advances in non-invasive imaging techniques have enabled cardiac function to be quantified during exercise thereby providing a novel means of identifying early cardiac dysfunction that has proved useful in several cardiovascular pathologies. The purpose of this narrative review is (1) to discuss the different non-invasive imaging techniques that can be used for quantifying different aspects of cardiac reserve; (2) discuss the findings from studies of cancer patients that have measured cardiac reserve as a marker of CTRCD; and (3) highlight the future directions important knowledge gaps that need to be addressed for cardiac reserve to be effectively integrated into routine monitoring for cancer patients exposed to cardiotoxic therapies.

## Introduction

Cancer-related survival has improved substantially over the past few decades due to advances in early detection and improved treatment regimens (1). This is resulting in a rapidly expanding population of cancer survivors, with more than 15.5 million cancer survivors in the United States alone (2). However, improvements in survival have unmasked an increased risk of morbidity and mortality from other chronic diseases (3–5). Of particular concern is an increased risk of cardiovascular disease, most notably amongst breast and hematologic cancer survivors (6, 7). This occurs due to a direct cardiovascular injury induced by a number of cancer treatments, alongside the indirect insults from common cardiovascular risk factors which can be exacerbated following a cancer diagnosis (8, 9). These cardiotoxic effects can present as hypertension, heart rhythm disorders, thromboembolic events, ischemic heart disease, cardiac dysfunction, and heart failure (8, 10). The development of cardiac dysfunction (and subsequent heart failure) following cancer treatment is of particular concern, due to its relatively high long-term incidence (cardiac dysfunction: 8–27%; heart failure: 2–10%) (3–5, 11) and poor prognosis (12). Therefore, the focus of this review is cancer treatment related cardiac dysfunction (CTRCD) with a specific attention toward comparing exercise measures of cardiac function with current standard-of-care measures performed in the resting state.

Cancer treatment related cardiac dysfunction can be broadly considered as abnormalities that develop in systolic or diastolic function as a result of exposure to cardiotoxic cancer treatments (8, 13). It is particularly common among patients exposed to anthracycline chemotherapy and incidental cardiac radiation, both of which have been associated with irreversible and dose-dependent cardiac dysfunction (8, 13, 14). There is also growing awareness that “targeted therapies” such as anti- human epidermal growth factor receptor 2 agents can also result in CTRCD (13, 15). These associations may explain why the rates of CTRCD are highest in breast cancer and hematological cancer survivors in whom combinations of cardiotoxic treatments are often prescribed (8, 13). However, many other cancer treatments can contribute to a patient's long-term risk of heart failure via their impact on key cardiovascular risk factors (9, 13). As such, an absence of measurable cardiotoxicity at the completion of treatment should not be taken as reassurance that a patient's risk of subsequent heart failure is low. Indeed, large prospective studies have shown that the risk of cardiac dysfunction and heart failure rises linearly, if not exponentially during survivorship (4, 7, 11, 16, 17). The disparity between acute cardiotoxicity and long-term risk of heart failure suggests that the current approach for detecting cardiotoxicity lacks the necessary sensitivity for identifying those who will go on to develop CTRCD. Importantly, whilst limited to evidence from two small single-center studies, prognosis for those who progress to symptomatic heart failure that is not detected until a number of years after cancer treatment is significantly worse than other forms of heart failure (12, 18). There is limited evidence demonstrating early initiation of therapy can improve heart failure outcomes, however, early detection of CTRCD with prompt initiation of heart failure therapies following cancer treatment has been associated with increased likelihood of recovery of cardiac function (19, 20). In contrast, patients among whom CTRCD was detected late showed limited recovery with heart failure therapy (19, 20). As such, there is growing interest in the development of strategies that can identify early stages of CTRCD when it may be more likely to respond to therapy. However, it is also important to note that there is limited evidence demonstrating that the prevention of early CTRCD translates into reduced risk heart failure in the long term. This highlights the need for long-term studies the trajectory of CTRCD to heart failure events, but also the need to provide a comprehensive assessment of cardiac function and heart failure risk that can account for transient changes in cardiac function that could result from acute side effects of cancer treatment such as anemia and hypotension.

## Current Approach for Detecting CTRCD

Currently, the cornerstone for detecting CTRCD is the quantification of left-ventricular (LV) systolic function using non-invasive cardiac imaging (10, 21, 22). The measurement of left-ventricular ejection fraction (LVEF) via two-dimensional (2D) echocardiography or radionuclide angiocardiography (RNA) is the current standard of care for detecting CTRCD in cancer patients (10, 21, 22). There is still no clear consensus for defining CTRCD, however the most-commonly used definitions describe an asymptomatic drop in LVEF by >10–15% points below a threshold ranging from 50 to 55%, or a drop of >5% points in the presence of heart failure symptoms (10, 21, 22). Although LVEF has been in use for decades, it has a number of limitations that have led some to question its ability to reliably detect the early stages of CTRCD (23). Firstly, the re-test variability in 2D echocardiography measures of LVEF is 10–13% (24–26), which, based on the definition of cardiotoxicity outlined above, severely limits its ability to detect what could be clinically important reductions in cardiac function. This limitation can be reduced using 3D echocardiography (variability of 5–6%) (25, 26), or perhaps also with RNA (variability reported to be as low as 1.5–5%) although, relative to echocardiography, there is a paucity of contemporary data interrogating the accuracy of RNA derived LVEF (27, 28). Furthermore, comparison to gold-standard CMR-derived measurements shows RNA may misclassify LVEF in cancer patients as either normal/abnormal in up to 20–35% of cases (29). Moreover, the significant radiation dose associated with RNA limits its use for serial examinations. In addition to issues with re-test reproducibility, there is growing awareness that measurement of LVEF is only sensitive to certain forms of cardiac dysfunction. Indeed, more than half of all heart failure cases now present as heart failure with preserved- (HFpEF), rather than reduced ejection fraction (HFrEF) (30), highlighting that relying on LVEF alone can result in a significant proportion of heart failure cases being missed. Finally, a reduction in resting LVEF is considered a relatively late outcome, as initial reductions in LV contractility can be compensated for by the remaining myocardium to maintain resting hemodynamics (31). Thus, there is growing interest in alternative tools for early detection of CTRCD such as myocardial deformation imaging and blood-based biomarkers of myocardial stress and injury (namely, troponin and b-type natriuretic peptide) (10, 23). There is particular emphasis on the use of LV global longitudinal strain (GLS) measurements as a more sensitive marker of LV dysfunction as it provides superior reproducibility to LVEF (32), and changes in GLS tend to precede reductions in LVEF following cancer treatment (32). Importantly, GLS has a superior ability than LVEF to predict cardiovascular outcomes in individuals with heart failure (33), and is able to predict the development of heart failure in individuals with heart failure risk factors (34, 35), and even in the general population (36). However, it is also important to acknowledge that whilst GLS has superior technical performance to LVEF assessment, and may be a superior predictor of subsequent declines in resting LVEF, neither of these parameters have been associated with the development, nor prognosis, of cancer treatment-induced heart failure (32). It should also be emphasized that the interpretation of changes in GLS in the short-term following cancer treatment may be confounded by changes in loading conditions (37–39) that can be highly variable during cancer treatment. Consequently, there are still a number of questions that need to be addressed before these measures can be widely incorporated into clinical care. This article will not provide a comprehensive overview of these techniques, as a number of excellent reviews already exist on this topic (23, 40). Rather, the aim of this review is to introduce an alternative paradigm for identifying sub-clinical CTRCD, which looks to assess cardiac reserve by measuring cardiac function during exercise, rather than at rest.

## Cardiac Reserve as a Marker of Cardiac Dysfunction in Heart Failure

**Figure 1:**
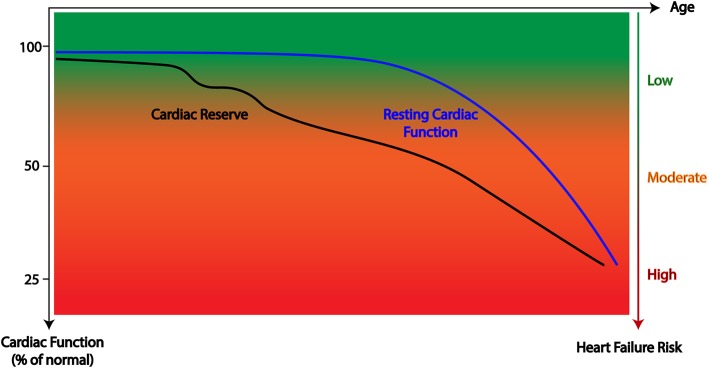
Graphical representation of the hypothesized trajectory of cardiac reserve and resting cardiac function following cancer treatment whereby reductions in cardiac reserve often precede measurable reductions in resting function.

One of the key limitations of the existing approaches for detecting CTRCD is that they assess cardiac function in a resting state, whereas symptoms of heart failure typically present on exertion. This introduces the concept of *cardiac reserve (defined as the increase in cardiac function from rest to peak exercise)*, which implies that the heart's functional capacity may be more completely determined by assessing its output under the hemodynamic and metabolic demands of exercise. Cardiac reserve assessment does not specifically relate to one parameter of cardiac function (stroke volume, LVEF, GLS), but the premise that measuring the response of these parameters to increased demand overcomes the limitations of the standard approach of measuring cardiac ejection and filling at rest, and trying to extrapolate what this performance will be like under increased demand when patients typically develop symptoms. Indeed, individuals with heart failure demonstrate a reduced augmentation of cardiac function during exercise, even in those who show relatively normal measures of resting cardiac function (41–47). As heart failure worsens, the ability to augment cardiac function during exercise becomes progressively impaired, and this coincides with increases in symptom severity and worsening exercise capacity [measured objectively by the peak oxygen consumption measured from a maximal cardiopulmonary exercise test [VO_2_peak]] (41, 46, 48). Importantly, evidence from a number of large prospective studies using indirect assessments of cardiac reserve such as VO_2_peak or peak metabolic equivalents (METs) measured in middle-aged and older adults without cardiovascular disease show strong, linear associations with risk of incident heart failure (well before resting measures of cardiac function are impaired) (49, 50), and are strong predictors of cardiovascular morbidity and mortality in individuals with established heart failure (51–53). Whilst there is a paucity of data among otherwise healthy individuals, there are also smaller cohort studies of individuals with cardiovascular pathologies such as heart failure, pulmonary hypertension, and heart transplantation, demonstrating specific measures of cardiac reserve during either exercise or pharmacologic stress provide a superior prediction over resting measures for subsequent cardiovascular morbidity and mortality (46, 54–59). As such, if these associations are also true for cancer survivors, the assessment of cardiac reserve may provide superior information on sub-clinical dysfunction and prognosis of the cardiac injury induced by cancer treatment (Figure 1). Intriguingly, despite often having preserved resting cardiac function (measured by LVEF), a reduction in exercise capacity is also a common finding in breast and hematological cancer survivors—many of whom were previously exposed to cardiotoxic cancer treatments—with VO_2_peak values reported as being 62–78% of normative values (60–65). Furthermore, there is growing evidence that impairments in VO_2_peak predict increased risk of all-cause and cancer-specific mortality in selected cancer populations (62, 65–67), but no studies have investigated whether this also predicts CTRCD or subsequent heart failure. However, whist reductions in VO_2_peak could be indicative of sub-clinical CTRCD, impairments in VO_2_peak could also result from peripheral impairments such as vascular and skeletal muscle dysfunction (68). This highlights the need for tools that can specifically assess cardiac function during exercise, with the most promising of these being exercise echocardiography, exercise RNA, and exercise cardiac magnetic resonance imaging (exCMR). In non-cancer populations, these techniques have been used to unmask cardiac dysfunction in individuals with exertional symptoms and normal or borderline resting cardiac function (such as HFpEF) (43, 44, 46, 47, 69). In this review we aim to (1) to provide a critical overview of the different non-invasive imaging techniques available for the quantification of cardiac reserve; (2) examine the findings from studies that have investigated the clinical utility of cardiac reserve as a marker of early CTRCD; and (3) highlight important knowledge gaps that need to be addressed for cardiac reserve to be effectively integrated into routine monitoring for cancer patients exposed to cardiotoxic therapies.

## Techniques for the Assessment of Cardiac Reserve During Exercise

At present there are a number of approaches for the assessment of cardiac reserve. The challenge has been to develop tools with sufficient accuracy to measure cardiac function when challenged by the greater respiratory drive and rapid heart rates encountered during exercise. The first descriptions of cardiac reserve were performed using invasive catheterization and direct ventriculography (70, 71). However, there has been a subsequent shift toward non-invasive techniques that can quantify cardiac reserve, resulting in a number of major advances in our understanding of the contribution of cardiac reserve to common cardiovascular pathologies (44, 69, 72–78).

Consequently, exercise-based cardiac imaging techniques may be useful for characterizing CTRCD. However, these techniques come with a number of advantages and disadvantages that need to be considered when determining their utility for detecting CTRCD (summarized in Table 1), These will be discussed in further detail in the following sections.

**Table 1:** Comparison of the strengths and limitations of the direct cardiac imaging techniques used during exercise.

**Technique**	**Spatial resolution**	**Temporal resolution**	**Simultaneous biventricular assessment**	**Repro-ducibility**	**Low cost and availability**	**Comments**
M-mode echocardiography	++	+ + +	+	+	+ + +	Optimal temporal resolution; significant geometric assumptions
Doppler echocardiography	NA	++	-	+	+ + +	Beat-to-beat assessment of cardiac output; no insight in regional myocardial motion; can provide assessment of diastolic parameters and pulmonary artery pressures
2D echocardiography	++	++	+	+	+ + +	Beat-to-beat assessment of global and regional cardiac motion; cardiac motion through imaging plane
Myocardial deformation imaging: Tissue Doppler and 2D speckle tracking	NA	++	+	+	+ + +	Quantitative assessment of global and regional cardiac deformation in multiple planes; technically difficult during exercise
3D echocardiography	+	+	+	+	++	Difficult acquisitions in many subjects; minimal experience with exercise
Radionuclide angiocardiography	-	-	++	+ + +	++	Separation of cardiac chambers (RV/LV and atria/ventricles) difficult; long acquisition times; radiation exposure
Cardiac magnetic resonance imaging	++	+	+ + +	+ + +	+	No geometric assumptions; time consuming analysis; experience limited to few expert centers

*-, minimal; + + +, maximal, NA, not applicable*.

### Echocardiography

Echocardiography is recognized as the primary non-invasive tool for the evaluation of cardiac reserve because of its low cost, lack of ionizing radiation, and ability to assess global and regional cardiac motion without the need for a physiological steady-state (79). Most often, exercise echocardiography is performed immediately after peak exercise on a treadmill or cycle ergometer, where the patient is transferred into a supine position for imaging (45, 79). Less commonly, images are acquired during exercise on an upright or semi-recumbent cycle ergometer (45, 79). A number of echocardiographic techniques can be used to assess different indices of systolic and diastolic function. These include M-mode echocardiography, Doppler echocardiography, 2D and 3D echocardiography, and myocardial deformation imaging. An overview of each of these techniques will be provided below.

#### M-Mode Echocardiography

One dimensional wall motion analysis, or M-Mode echocardiography, offers excellent temporal resolution, and LV function can be measured as fractional shortening, which represents a simple measure of the percent change in LV diameter from end-systole to end-diastole. However, the accuracy of fractional shortening depends upon alignment of the ultrasound beam through the maximal diameter of the LV and then relies on extrapolation from one-dimension to a three-dimensional structure using the Teichholz formula (80). Consequently, it is no longer the echocardiographic technique of choice.

#### Doppler Echocardiography

**Figure 2:**
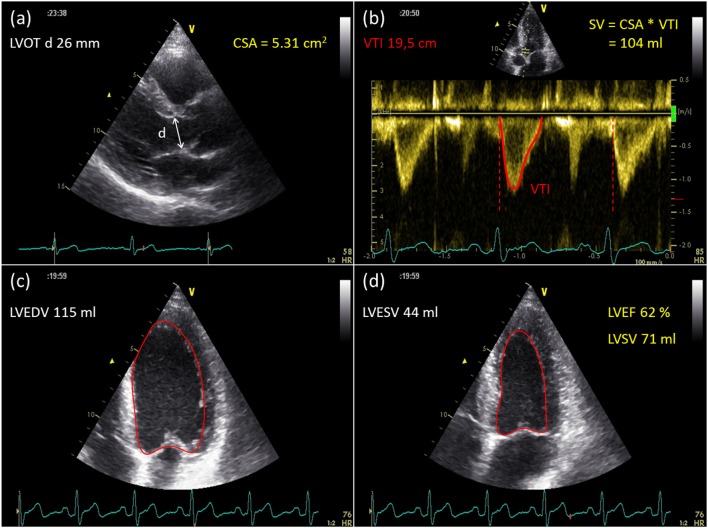
Example of stroke volume assessment with Doppler and 2D echocardiography. Using Doppler echocardiography, SV is calculated as the product of the cross-sectional area (CSA) of the LV outflow tract **(a)** and the velocity time integral (VTI) of flow through that area **(b)**. **(c,d)** Illustrate endocardial border delineation in a single plane four chamber view with 2DE. SV and EF are calculated with the summation of disks method, using geometrical assumptions.

In pulse wave Doppler echocardiography, ultrasound pulses are transmitted in a single line. The returning Doppler signal is displayed as a spectral curve of flow velocities against time with the area under the curve, i.e., the velocity time integral (VTI), representing the amount of flow passing through a given area which can be used to calculate stroke volume (SV; Figure 2), which can be multiplied by heart rate (HR) to calculate cardiac output (CO). Most commonly, the area of the LV outflow tract (LVOT) is derived from measures of LVOT diameter and combined with VTI estimates taken at the same level. Another key feature of Doppler echocardiography is its ability to provide an insight into key aspects of diastolic function using pulse-wave and tissue Doppler imaging.

Pulse-wave Doppler imaging can also be used to assess diastolic function at rest and during exercise. The pattern of trans-mitral inflow velocities during early and late diastole can be combined with tissue Doppler imaging of septal and lateral mitral annulus velocities during early diastole (e') to provide an estimate of LV filling pressures (81, 82). A handful of small to moderately sized studies (*n* = 10, 12, and 75) of patients with HFpEF and/or exertional dyspnoea have shown that compared to resting Doppler assessment, the inclusion of E/e' >13–15 during exercise had a sensitivity of 67–90% for detecting an abnormal increase in LV filling pressures or pulmonary capillary wedge pressures (82–84). Whilst showing promise, there are concerns about the ability of E/e' to reliably track changes in LV filling pressures at the individual level. For example, whilst Talreja et al. (84) found a significant correlation between changes in E/e' and pulmonary artery pressure at the group level, there was substantial variability in this relationship at the individual level. Furthermore, Maeder et al. (85) also demonstrated no change in E/e' during exercise despite a significant increase in pulmonary artery pressure. This highlights the need for further validation of exercise E/e' as a marker of diastolic reserve and LV filling pressure.

The assessment of tricuspid regurgitant velocities using Doppler imaging can be used to estimate changes in systolic (sPAP) and mean pulmonary artery pressures (mPAP), and thereby identify impairment in pulmonary vascular and diastolic reserve. Evaluating the slope of the increase in sPAP relative to CO during exercise is considered a reasonably accurate marker of impaired pulmonary vascular reserve (86). A review of invasive and imaging-based studies has shown that slope values above 3 mmHg/L/min are consistently associated with pulmonary vascular pathology (86). Although this has traditionally been assessed with invasive pressure measurements, our group has shown that this can be accurately assessed using Doppler echocardiography measurements of sPAP and CO during semi-supine cycling (75). Whilst there have been some questions as to the feasibility of Doppler-derived assessment of tricuspid regurgitant velocities and sPAP during exercise (83), this can be dramatically improved with the use of colloid-agitated contrast enhancement (69, 87, 88). Furthermore, an advantage of the sPAP/CO slope measurement is that it does not require a peak exercise value, as it can be calculated from multiple measures obtained during sub-maximal exercise (which are less technically demanding).

#### 2D Echocardiography

2D echocardiography provides cross-sectional images of the heart. Integration of image slices from different viewing orientations enables measurement of ventricular dimensions and appreciation of global as well as regional myocardial function. Using 2D echocardiography, ventricular volumes and ejection fraction can be determined using the biplane method of disks, applied on the apical 4- and 2-chamber view (modified Simpson's rule). To facilitate data acquisition during exercise, ventricular volumes can also be determined from a single plane 4-chamber view (Figure 2), with similar accuracy to those calculated by the biplane method (at least in normal subjects with relatively symmetrical ventricular geometry) (89). Another method to calculate LV volumes during exercise is the area-length method, which involves measurement of the mid-LV cross-sectional area from the parasternal short-axis view and the ventricular long-axis length from annulus to apex in the apical 4-chamber view (90). However it should be noted that 2D echocardiography provides a number of challenges for accurate image acquisition due to issues with motion artifact and image foreshortening. For individuals with poor endocardial delineation, the administration of contrast agents can significantly improve the accuracy of endocardial border detection (91, 92) and calculation of LV volumes at rest (93, 94) and during exercise (92, 95).

#### 3D Echocardiography

3D echocardiography has some important theoretical advantages over 1D and 2D approaches for chamber visualization and quantification. The rapid acquisition of pyramidal datasets eliminates the need for geometrical assumptions and errors caused by foreshortened views. However, given that a full volumetric data acquisition is typically performed over at least 4–7 cardiac cycles, so-called “stitching artifacts” may appear between sub-volumes as a result of cardiac translation due to respiration. The recent development of single-beat 3D echocardiography should avoid these stitching artifacts, but, at present, image quality is limited. Whilst showing improved accuracy and repeatability over 2D echocardiography at rest (96, 97), currently, there is a lack of studies validating the use of 3D echocardiography for the assessment of cardiac volumes during exercise (either in healthy or clinical populations), which is likely related to the challenges in obtaining images with adequate temporal resolution at high heart rates.

#### Myocardial Deformation Imaging

**Figure 3:**
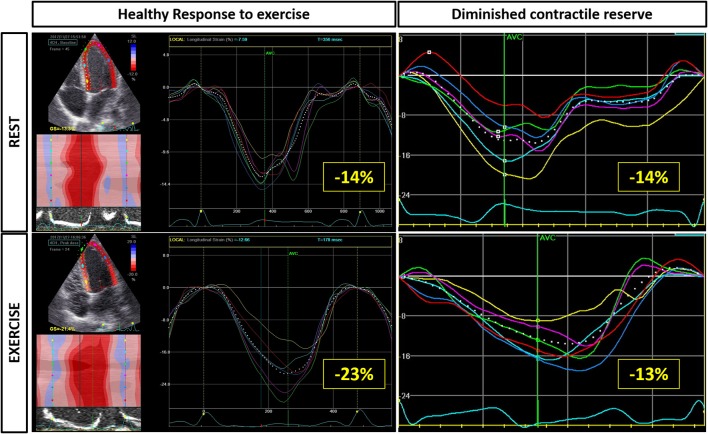
Deformation imaging with exercise can provide insights into subtle cardiac dysfunction. In this example, resting longitudinal strain assessed by 2D speckle tracking echocardiography is reduced in both patients. However, in the healthy heart strain increases from −14 to −23% but in the heart with abnormal contractility the strain does not augment with exercise. Furthermore, note that in the abnormal heart, the peak strain becomes progressively more delayed.

One of the major constraints of using cardiac volume measures, such as LVEF, to assess myocardial performance is that these measures are strongly influenced by loading conditions. Measures of myocardial velocity and deformation (strain and strain rate) have gained increasing acceptance as accurate and reproducible measures of myocardial function. Assessments of GLS and strain rate during exercise has shown the ability to discriminate between athletic and sedentary individuals (98), identify cardiac impairment due to aortic stenosis (99) or cardiac allograft vasculopathy following heart transplantation (100), and can predict individuals at increased risk of cardiac events following heart transplantation (55). The high temporal resolution of tissue velocity imaging (TVI) enables measurement of brief events such as the isovolumic acceleration, which is suggested to be a relatively load independent measure of contractility that can be measured during exercise (101, 102). Strain rate has been assessed in several studies as a surrogate measure of LV and right-ventricular (RV) contractility during exercise (102, 103). 2D speckle tracking echocardiography (STE) is a non-Doppler technique which relies on tracking of ultrasound speckles within the image (104). Given its angle-independency, STE also allows for appreciation of LV and RV GLS (104). The assessment of LV and RV peak strain and GLS during exercise may provide an insight into contractile reserve (thereby overcoming the theoretical limitations of resting measurements—Figure 3). Whilst posing additional technical challenges, exercise strain assessment using TVI and 2D speckle-tracking echocardiography has adequate feasibility and reproducibility compared to other echocardiographic techniques (98, 103). 2D STE has superior reproducibility than TVI (102, 105), however this comes at the cost of an increased reliance on image quality (104), which can be problematic during exercise. Consequently, feasibility and accuracy of 2D STE measurements is markedly reduced above moderate exercise intensities (102, 105), primarily due to inadequate frame rate at high heart rates. The higher temporal resolution of TVI makes it the preferred candidate for assessing changes in strain rate during high intensity exercise, however the higher variability of TVI may limit its use at the individual patient level.

#### Comparison of Echocardiographic Techniques

The most obvious question to arise after discussing the different echocardiographic techniques is: which is now the best echocardiographic method to assess cardiac volumes and function during exercise? The most commonly used echocardiographic techniques to assess SV and CO are Doppler echocardiography and 2D echocardiography. Christie et al. (89) compared the accuracy of Doppler echocardiography and 2D echocardiography for assessing CO during exercise against Fick oximetry and thermodilution. Correlations with invasive measures were reasonable with the Doppler method (SV: r = 0.66–0.67; CO: r = 0.78–0.81) and could be obtained in all participants. Better correlations were obtained by 2D echocardiography (SV: r = 0.72–0.81; CO: r = 0.88–0.90), but measures could only be obtained in 3 of 10 participants at peak exercise and underestimated invasive values by approximately 60% (89). Compared with 2D echocardiography, pulse wave Doppler echocardiography is limited by the fact that it only measures SV rather than changes in biventricular volumes during diastole and systole. In contrast, 2D echocardiography is limited by the need for geometric assumptions and difficulties standardizing the imaging plane due to through-plane cardiac translation resulting from increased respiration. These problems are even more pronounced for assessment of RV volumes during exercise due to the complex geometry of this chamber, difficulty in accurately delineating the endocardium due to its pronounced trabeculation, and its retrosternal position, which limits echocardiographic imaging windows. Finally, myocardial deformation imaging can provide a unique insight into the myocardial systolic and diastolic response (and their interactions), however this can be very technically demanding (particularly during high intensity exercise), and there is limited data on the accuracy of different vendor platforms for assessment during exercise. It is also important to note that cancer-specific considerations such as chest-targeted radiation, breast surgery may result in poor acoustic windows for echocardiographic assessment.

### Radionuclide Angiocardiography

Radionuclide angiocardiography is a nuclear imaging technique that can evaluate ventricular performance during exercise. Most often, imaging is performed during supine or upright exercise on a bicycle ergometer. Radionuclide angiocardiography can be differentiated into two key techniques: (1) first-pass radionuclide angiocardiography (FPRNA) and (2) equilibrium radionuclide angiocardiography (ERNA).

FPRNA comprises a rapid bolus injection of radiotracer, preferably Technetium99m diethylamine triamine pentaacetic acid because of its renal excretion, hence minimizing radiation exposure (106). After bolus injection, radioactivity is measured during the first passage of the tracer through the heart. Generation of time-activity curves allows determination of LV end-diastolic and end-systolic counts, from which SV, EF, and CO can be inferred. ECG gating is required (especially for single-crystal gamma cameras) to allow reconstruction of 25–50 ms frames to calculate an EF. Ventricular volumes can be measured by a geometric or count-proportional technique. Similar to the dye-dilution methods, CO can also be assessed more directly by measuring the area under the time-activity curve. FPRNA provides temporal separation of the cardiac chambers with high target-to-background ratio, as radioactive counts are accumulated only during the initial passage of the isotope as it traverses the central circulation. Therefore, the radioactive bolus will have been ejected from the RV before entering the LV. This technique can be applied whilst exercising, but only in one projection, usually the anterior for LV function studies. For assessment of RV function, preferably a shallow right anterior-oblique view is recommended (106). Typically, data acquisition requires 20–50 s. Because of these relatively short acquisition times, FPRNA can be performed at high exercise levels (107).

**Figure 4:**
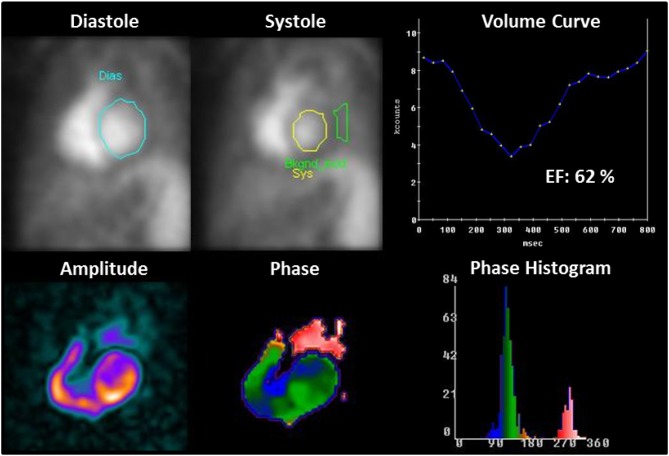
Example of LV function assessment with equilibrium-gated radionuclide angiocardiography. After drawing a region of interest (ROI) at end-diastole (blue contour) and end-systole (yellow contour), a complete LV volume curve or time-activity curve can be generated. The measured LV counts within these LV ROIs are corrected for background activity (BkCorr), which is measured using a ROI adjacent to the end-systolic border (green contour). LVEF is then calculated using the equation: [(BkCorr end-diastolic counts – BkCorr end-systolic counts)/BkCorr end-diastolic counts] × 100. Phase and amplitude analysis provide quantitative information about regional wall motion.

In ERNA, the radioactive tracer is attached to patients' red blood cells or human serum albumin and allowed to reach equilibrium in the vascular system before imaging is performed (108). Image acquisition is triggered by the R-wave on the electrocardiogram such that the duration of each cardiac cycle can be identified as the interval between two successive R-waves. Each of the 200–600 cardiac cycles are summed and divided into 16–32 frames. Corresponding frames of multiple cardiac cycles are summed together during an acquisition period of at least 3 min. Therefore, in contrast to resting ERNA studies, acquisition time is often the limiting factor during exercise. However, previous research has demonstrated the feasibility of ERNA during peak exercise using short acquisition times (109). A time-activity curve is generated within a region of interest, involving the LV, to display the change in radioactive counts throughout the cardiac cycle (Figure 4). The peak and nadir of the time-activity curve represent the end-diastolic and end-systolic counts, respectively. Measurement of ventricular volumes using ERNA is susceptible to a number of problems. First, accurate assessment of ventricular volumes using ERNA requires adequate separation of cardiac chambers. Because of the considerable overlap between the RA and the RV, ERNA is not appropriate for evaluation of RV volumes or function. Furthermore, successful gating relies on regular heart rates, such that ERNA cannot be performed in patients with marked arrhythmias.

An important advantage of RNA techniques is that measurements of ventricular EF are not dependent on geometrical assumptions, but are based on the assumption that LV volumes are proportional to LV counts throughout the cardiac cycle. Ventricular volumes can be derived by a counts-based or geometrically based method, but these measurements are not routinely performed in a clinical setting. RNA techniques provide very reproducible measurements of EF both at rest and during incremental exercise (110, 111). Importantly, EF can be obtained in virtually all patients, regardless of body habitus, during maximal exercise (109, 111). However, one of the main limitations of RNA compared to cardiac magnetic resonance imaging (CMR) and echocardiography is its radiation exposure. This may be particularly important in populations who require ongoing cardiac monitoring such as childhood and adolescent cancer survivors. Furthermore, due to its intrinsic low spatial resolution, RNA contributes little to the characterization of anatomical structures or pressure gradients—both of which may also be important markers of CTRCD.

### Cardiac Magnetic Resonance Imaging

**Figure 5:**
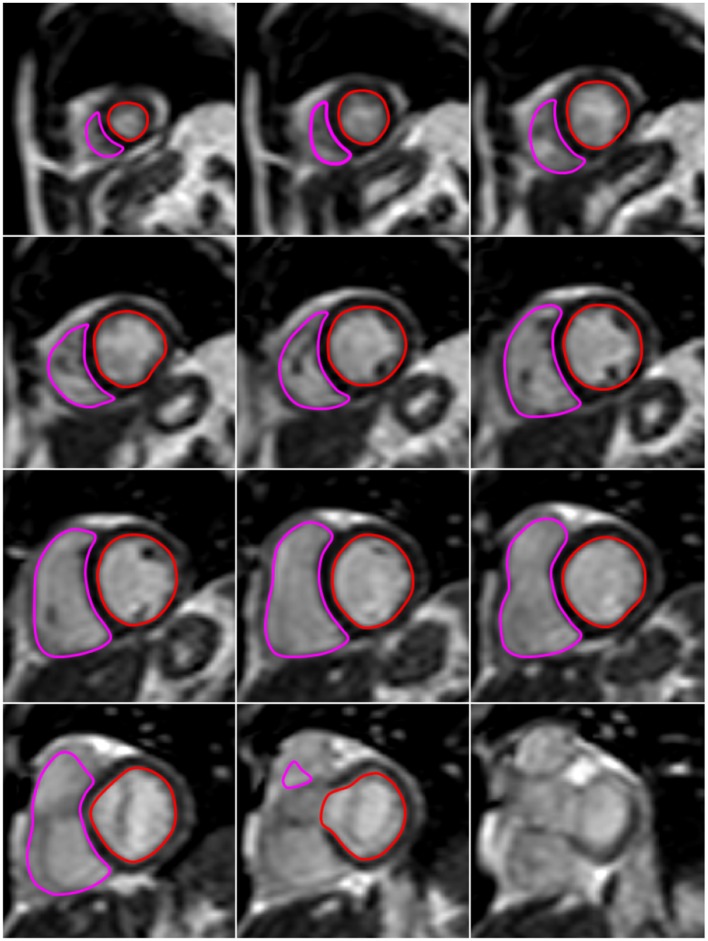
Example of biventricular volume analysis with exercise CMR. The images presented in this figure are end-diastolic image slices in the short axis that cover the heart from apex (upper left image) to base (lower right image). At all slice levels, LV and RV endocardial borders are delineated in red and pink, respectively. The LV and RV volume within each slice is derived as the area within the red and pink contours, respectively, times slice thickness. For each ventricle, the total end-diastolic volume is calculated as the sum of the volumes of all slices from apex to base. Similarly, LV and RV end-systolic volume is the sum of the slice volumes obtained at end-systole (not shown here).

CMR is generally considered the gold standard technique for measurement of biventricular volumes and cardiac function at rest (22). Rather than depending on single image slices and multiple geometrical assumptions, the entire heart can be sampled without limitations in acoustic windows. Typically, CMR requires electrocardiographic gating and multiple breath holds. For each frame in the cardiac cycle, an image is acquired over several heartbeats. All acquired image frames can be displayed as a cine loop, with optimal spatial and temporal resolution. To measure RV and LV volumes, a stack of short-axis slices covering the ventricles from base to apex is acquired. Each slice contains a cine loop of image frames throughout the cardiac cycle. Endocardial borders from end-diastolic and end-systolic frames can be contoured in a manual or automated manner and volumes for both ventricles can be calculated using the slice summation method (Figure 5). The acquisition of a complete set of cine CMR images can be performed in 5–10 breath-hold periods of 10 s. However, the need to perform multiple breath holds and the relatively long acquisition times, limit the use of cine CMR imaging in exercise testing. Furthermore, the ECG signal is distorted by the magnetic field of the CMR system because of the magnetohydrodynamic effect which makes it extremely challenging to acquire reliable cardiac gating during the increased movement and blood flow associated with exercise.

To overcome these problems, real-time exCMR imaging has been developed (112, 113). In this mode, each image frame of the cardiac cycle is acquired independently and sequentially. Using techniques such as parallel imaging to improve temporal resolution, a complete image frame can be obtained within 35 ms, with only a slight compromise in spatial resolution. Because of such rapid data acquisition, the effects of cardiorespiratory motion are less pronounced and it becomes possible to visualize the beating heart during free breathing and without cardiac gating (112). As a result, real-time exCMR can be used to assess biventricular volume changes throughout exercise, without the need for geometrical assumptions or radiation exposure (112). A comparison with direct measurement (via catheterization) of cardiac function among athletes, arrhythmia patients and individuals with heart failure suggests exCMR is capable of measuring cardiac function with a high degree of accuracy (R = 0.96 for CO), precision (inter-observer CV for stroke volume: 1.9–5.0%) and reproducibility (Intra-class correlation coefficients: 0.86–0.94) (113). Simultaneous assessment of both ventricles also enables the study of ventricular interaction during exercise. This may be important as there is limited data regarding the RV response to exercise, and evolving evidence that it may be of importance to overall cardiac performance in health and disease (78, 114). Furthermore, the RV can also be impacted by cancer treatment such as anthracyclines (115, 116), however current research is primarily focused on the LV. Tools such as exCMR, which can provide a detailed quantification of combined LV and RV function may better elucidate the influence of cardiotoxic cancer treatments on RV function (and its interaction with LV function).

Another CMR method for determination of SV is CMR phase-contrast flow quantification (117). This technique uses application of bipolar magnetic field gradients to encode the velocity of moving blood. First, a region of interest is delineated around the vessel lumen to determine the cross-sectional area of the vessel. The spatial mean velocity for all pixels in this area is measured and plotted against time. The instantaneous flow volume across the vessel lumen can be calculated by multiplying the spatial mean velocity by the cross-sectional area. Finally, integrating the instantaneous flow volumes for all frames throughout the cardiac cycle yields the total flow volume per heartbeat. Systemic and pulmonary blood flows can be measured from measurements in the proximal ascending aorta and pulmonary trunk, respectively. However, similar to pulse wave Doppler echocardiography, CMR phase-contrast flow quantification is susceptible to aliasing if the flow velocities are too high. An advantage of CMR phase-contrast vs. Doppler echocardiography is the fact that it is not limited by acoustic windows. However, phase-contrast quantification is very dependent upon maintaining a stationary imaging plane and the respiratory translation and whole body movement during exercise can result in considerable error. Nevertheless, some groups have shown that this technique is feasible (118–121), and have demonstrated high correlations for exercise SV (R = 0.988) measured using real-time phase-contrast quantification and real-time assessment LV volumes (122).

Despite several important advantages over other imaging techniques, CMR also has its limitations. The key disadvantages of exCMR are its high cost and limited availability. Furthermore, exercise imaging with CMR is confined to exercise in the supine body position. Usually, this is performed on a supine cycle ergometer attached to the CMR table, which allows for in-magnet exercise testing. Some groups have used treadmill exercise outside the scanner to achieve maximal cardiovascular stress, followed by post-exercise breath-hold cine imaging (123). However, the rapid changes in physiology during recovery may render this a poor surrogate of real-time exercise values. Open-bore CMR systems might enable cardiac volume assessment during upright exercise in the future. Cheng et al. (124) demonstrated the feasibility of CMR phase-contrast flow quantification during upright exercise in an open CMR magnet. However, compared with conventional CMR scanners, open CMR scanners typically have a lower magnetic field strength, which compromises image quality and prolongs acquisition times. Currently, volumetric assessment of the heart using real-time CMR has not been performed during upright exercise.

### Pharmacological Stress

Pharmacologic stress can be an alternative means of assessing cardiac reserve among patients who may be unable to exercise. This has primarily been used for the assessment of inducible ischemia and myocardial viability, either through inotropes such as dobutamine, or vasodilators such as adenosine. Dobutamine induces β_1_-mediated increases in heart rate and contractility, and β_2_-mediated peripheral vasodilatation, thereby simulating some of the physiologic changes seen during exercise. An advantage of pharmacologic stress over exercise imaging is the improved image quality due to lack of movement. The utility of pharmacologic assessment of cardiac reserve in the heart failure setting has primarily been investigated by stress echocardiography, with studies investigating the utility of pharmacologic stress CMR primarily limited to the diagnosis and prognosis of coronary artery disease (125, 126). Cardiac reserve, assessed by changes in contractility (either as EF or wall motion score index) measured during dobutamine echocardiography in patients with established heart failure has been associated with heart failure prognosis (127, 128) and response to medical therapy (129, 130). However, it is unclear whether pharmacologic assessments of cardiac reserve can identify at risk individuals who will go on to develop heart failure. Furthermore, it is important to note that whilst dobutamine induces physiological changes that are similar to exercise, these techniques should not be considered interchangeable. Compared to exercise, dobutamine infusion has been associated with a blunted increase in stroke volume, cardiac output and LV wall stress (131)—likely due to disparate effects on preload and afterload between the two techniques. Consequently, where possible, exercise should be the preferred modality given it is more likely to induce the physiology where heart failure symptoms such as fatigue and exertional breathlessness occur. This decision making should also consider the potential risks from pharmacologic stress such as arrhythmias, nausea, dizziness and vomiting.

### Additional Considerations

There are also general considerations related to the assessment of cardiac reserve that warrant discussion. The type and timing of exercise are important as they can also influence the sensitivity for detecting cardiac dysfunction. These may be dictated by equipment availability, patient familiarity and the imaging modality being used (79). The pattern and type of muscle recruitment during treadmill exercise is often more familiar to patients than bicycle exercise, which improves the ability of the patient to achieve legitimate cardiovascular endpoints (as patients may be limited by peripheral muscle fatigue during cycling). Furthermore, a number of childhood cancer patients may be too small to effectively use a stationary bicycle, and so a treadmill may be the only method available for conducting exercise testing. However, acquiring images *during* walking or jogging using echocardiography is very technically demanding, and is not possible using existing CMR or nuclear imaging techniques. Therefore, it is really only feasible to perform imaging *immediately following* the exercise bout if treadmill is the preferred mode of assessment. This is an important consideration as cardiac loading can quickly return to normal following exercise (132), and so subtle dysfunction may be missed during the post-exercise image acquisition period. An advantage of supine or semi-supine bicycle ergometer is the ability to assess cardiac function *during progressive exercise*, and so cardiac function can be directly compared to the development of a patient's exertional symptoms (79). However, cycling in the supine position is uncomfortable and there are physiological differences to upright exercise that mean the maximal workloads and heart rates achieved with supine exercise are usually lower than in the upright position (133, 134). It is important to acknowledge that cardiac reserve assessment requires additional time, equipment, and expertise beyond standard resting cardiac function assessments. However, whilst being more demanding than resting assessment, there is potential for improved patient outcomes and subsequent cost savings if cardiac reserve proved to have additional predictive value over resting measures. Given the aim of continually improving patient care and the increasing importance of cardiovascular health in cancer survivors, there is a strong rationale for pursuing further research into the potential cost-effectiveness of measures of cardiac reserve. The timing of cardiac reserve assessment in relation to cancer treatment is another important consideration. There are no studies to guide the optimal timing of cardiac reserve assessment in the oncology setting, however following the standard guidelines for other assessments would be a reasonable starting point (10). Timing of assessment should consider the type, length, frequency, and dose of treatment, in addition to each patient's baseline cardiovascular risk profile and self-reported symptoms (10). It would be reasonable to conduct assessment of cardiac reserve prior to treatment, which may aid in identifying patients with pre-existing dysfunction, whilst also providing a baseline measurement from which to compare subsequent results. An assessment at the completion of standard doses of anthracycline-containing chemotherapy would also be logical, whilst further assessment may be needed for patients with increased risk of heart failure such as those scheduled to receive high doses of chemotherapy or other subsequent cardiotoxic treatments, or those for whom cardiac function is borderline. Testing too frequently may be problematic, and whilst treatment such as anthracycline chemotherapy is associated with dose-dependent cardiac injury it is unclear how long an interval is required for this to manifest into measurable dysfunction. This highlights the need for further work focusing on this question.

## The Utility of Cardiac Reserve in Cancer Patients Exposed to Cardiotoxic Therapies

### Pediatric Cancer Survivors

There are a number of studies (primarily cross-sectional) comparing cardiac reserve in pediatric cancer survivors treated with cardiotoxic therapies, largely anthracycline chemotherapy, to matched healthy controls (135–145). Compared to control subjects, blunted increases in LVEF or fractional shortening (135–137, 140, 146), attenuated increases in stroke volume and cardiac index (138, 141, 142, 144) or a blunted increase in LV longitudinal and circumferential strain (147) have all been reported. Another important question regarding the use of cardiac reserve in cancer patients is its ability to detect sub-clinical dysfunction. Unfortunately, the degree to which reductions in cardiac reserve precede impairments in resting function remain unclear, with some studies reporting resting function to be preserved (136, 137, 140, 142, 146, 147), whilst others report simultaneous reductions in resting function (138, 141, 144). This discrepancy between studies could be explained by the cross-sectional designs, differences in inclusion and exclusion criteria between studies, and the long duration since the completion of cancer treatment.

There are two studies that prospectively evaluated changes in exercise cardiac reserve among pediatric cancer survivors previously treated with anthracyclines (143, 145), however these studies have conflicting findings. Guimaraes-Filho et al. (145) found that whilst cancer survivors who were ~3 years from treatment completion had reduced exercise LVEF and increased end-systolic wall stress compared to control subjects, there was no change in cardiac function over the following 5 years (145). This makes it difficult to determine the benefit of longitudinal evaluation of cardiac reserve in this population. Sieswerda et al. (143) performed exercise echocardiography in 92 asymptomatic childhood cancer survivors 8.2 years post-chemotherapy, and whilst fractional shortening and LV end-diastolic dimension declined over the following 10 years of follow-up, the addition of baseline exercise fractional shortening measures did not provide additive benefit for predicting declines in fractional shortening beyond age, anthracycline dose and resting fractional shortening at baseline (143). Whilst these findings suggest cardiac reserve does not provide incremental value beyond resting measures in predicting future cardiac dysfunction, these findings should be interpreted with caution as exercise echocardiography was performed a number of years following treatment completion and only the most basic echocardiographic measures were employed.

The potential role of dobutamine stress has been investigated in several studies of anthracycline-treated pediatric cancer survivors. Cross-sectional studies of asymptomatic pediatric cancer survivors assessed with dobutamine stress echocardiography a number of years following anthracycline treatment (median 5.3–7 years) have shown cancer survivors have reduced posterior LV wall thickening, LVEF, and LV fractional shortening, decreased ratio of early to late peak mitral velocity (E/A), and increased LV wall stress during exercise compared to matched control subjects—demonstrating impairments in both systolic and diastolic reserve (148, 149). Furthermore, the degree of impairment in cardiac reserve appears to relate to the dose of chemotherapy received (150). In contrast, Lanzarini et al. found no differences in cardiac reserve in long-term (mean 7 years post-treatment) pediatric cancer survivors and matched sibling controls during low dose dobutamine stress echocardiography. One reason for this discrepancy could relate to differences in population characteristics, dobutamine infusion protocol and also the use of m-mode measurements, which as discussed previously, are limited by high variability. Furthermore, as these impairments are measured years following cancer treatment, a number of impairments in resting cardiac function were also present in a significant proportion of the cancer survivors assessed. Further prospective studies, using more contemporaneous measures of cardiac function are needed to better understand the trajectory and clinical significance of these differences.

Overall, it appears that there is sufficient evidence to suggest cardiac reserve is reduced in pediatric cancer survivors previously treated with cardiotoxic treatments (primarily anthracyclines), however more evidence is needed to prospectively evaluate changes in cardiac reserve following treatment and whether this can predict future CTRCD.

### Adult Cancer Survivors

Compared to pediatric cancer survivors, there are few reports investigating changes in cardiac reserve among adult cancer patients and survivors. Kirkham et al. (151) performed a systematic review (which included 14 studies) investigating the role of stress testing for the early detection of cardiovascular disease in breast cancer survivors. However, the majority of studies focused on the role of stress imaging (such as myocardial perfusion imaging) or stress electrocardiography for the early detection of coronary artery disease, rather than the prediction of CTRCD. Two cross-sectional studies (*n* = 30–57) included in this systematic review compared cardiac reserve measured via 2D echocardiography among early stage breast cancer survivors previously treated with anthracyclines to age- and gender-matched controls (152, 153). Whilst none of the breast cancer survivors met the criteria for cardiotoxicity determined by resting LVEF or global longitudinal strain, both studies found that breast cancer survivors had reduced cardiac reserve. Specifically, this included a lower LVEF at peak exercise (153), or a lower cardiac index and SV index at peak exercise (152). Recent work from our laboratory has shed some light on the evolution of cardiac reserve impairment in breast cancer patients undergoing anthracycline chemotherapy using exCMR (154, 155). In this series of studies, we found that exercise stroke volume tended to be lower shortly following anthracycline chemotherapy (*P* = 0.06) (155), however over the following 12-months this evolved into a blunted augmentation of LVEF and SV during exercise, resulting in a reduction in peak exercise CO (154). One of the more intriguing findings comes from a small (*n* = 48) prospective analysis of mixed adult and pediatric patients with advanced cancer undergoing high-dose anthracycline chemotherapy (156). This study assessed cardiac reserve using resting and exercise RNA LVEF prior to-, after completion of low-dose and high-dose anthracycline chemotherapy. Both resting and peak LVEF declined progressively during chemotherapy, with a significantly blunted increase in LVEF from rest to peak exercise at completion of low- and high-dose chemotherapy. Notably, four patients developed congestive heart failure 1–6 months following treatment, all of whom had normal pre-treatment resting LVEF, but an abnormal increase in LVEF during exercise (<4% increase), suggesting pre-treatment cardiac reserve may be a predictor of chemotherapy-induced heart failure. However, given the small numbers and high dose of treatment, this requires further validation in larger cohorts, and with chemotherapy does that would be more applicable to the broader population of adult cancer survivors.

There have also been a handful of studies investigating the role of pharmacologic stress to assess cardiac reserve in adult cancer survivors. Civelli et al. (157) investigated the prospective changes in resting and dobutamine stress LVEF throughout and shortly following anthracycline chemotherapy, and ability for resting and peak LVEF to predict late CTRCD 18-months following chemotherapy in 49 women with advanced breast cancer undergoing high-dose chemotherapy. When subjects were differentiated into those with normal (>50%) or impaired (<50%) resting LVEF at 18-months, it was found that whilst resting LVEF was similar at all previous time points, peak stress LVEF was significantly lower from the third cycle of anthracycline chemotherapy onwards. Importantly, a >5 point reduction in LVEF reserve was a significant predictor of impaired resting LVEF at 18-months. Cottin et al. (158) assessed changes in cardiac reserve using low-dose dobutamine stress echocardiography in a mixed population of adult cancer patients undergoing standard doses of anthracycline chemotherapy. These authors found that peak LVEF did not change from baseline to the end of chemotherapy, however at the end of chemotherapy there was a blunted increase in peak mitral flow velocity (E wave) and a decrease in the ratio between peak early and late mitral flow velocities (E/A) which may be indicative of poor diastolic reserve. The lack of change in peak LVEF may reflect the lower chemotherapy doses and shorter follow-up used in this study (158) compared to that of Civelli et al. (157). It would be interesting understand whether the subtle diastolic impairment demonstrated by Cottin et al. (158) evolves into more significant dysfunction over the months following therapy. In contrast, Bountioukos et al. (159) found no additional value of contractile reserve assessed by dobutamine stress echocardiography for predicting RNA-assessed reductions in LVEF among adult patients with hematological cancers. However, it should be noted that contractile reserve in this study was quantified using the change in wall motion score index, which is a relatively blunt method for quantifying cardiac reserve, and may lack the necessary sensitivity for assessing subtle changes in peak cardiac function in this setting.

Overall these results suggest cardiac reserve is impaired in adult cancer survivors treated with anthracyclines, and may be a more sensitive marker of subclinical dysfunction given resting function was often relatively preserved. However, due to the relatively small sample sizes, these findings require validation in larger cohorts, which also investigate their potential relationship with other important cardiovascular and health outcomes. Furthermore, the utility of cardiac reserve to detect cardiac dysfunction induced by other cardiotoxic cancer therapies requires further evaluation.

## Conclusions

Cardiovascular conditions are emerging as a major cause of morbidity and mortality among cancer survivors. Consequently improved detection and treatment of CTRCD can have a profound impact on health outcomes following cancer treatment, however the current approach for identifying CTRCD is largely insensitive to early cardiac dysfunction. The use of non-invasive imaging techniques to assess cardiac function during exercise enables a more wholistic characterization of cardiac function, and may therefore provide a novel means of early identification of CTRCD. Whilst there is emerging data demonstrating patients previously exposed to cardiotoxic chemotherapy and/or radiotherapy have impairments in cardiac reserve (despite relatively preserved resting cardiac dysfunction), there are still a number of gaps that should be addressed before this approach can be incorporated into the clinical setting. There is a need for longitudinal studies to better understand the evolution of impairment in cardiac reserve, and its relationships with other cardiovascular endpoints. Furthermore, given the variety of imaging methods and measures available for quantifying cardiac reserve, there is a particular need to identify the optimal imaging method, measure, and associated cut-points for predicting these outcomes. Ultimately, we are hopeful that existing and new imaging modalities focused on assessing cardiac function during exercise will help to unravel many unresolved questions, ultimately improving morbidity and mortality outcomes for the growing cancer survivor population.

## Author Contributions

All authors contributed to the conception, development, and critical appraisal of this manuscript.

### Conflict of Interest

The authors declare that the research was conducted in the absence of any commercial or financial relationships that could be construed as a potential conflict of interest.

## Abbreviations

CTRCD: Cancer treatment related cardiac dysfunction
LV: Left ventricular
LVEF: left ventricular ejection fraction
2D: two-dimensional
RNA: radionuclide angiocardiography
3D: three-dimensional
HFpEF: heart failure with preserved ejection fraction
HFrEF: heart failure with reduced ejection fraction
exCMR: exercise cardiac magnetic resonance imaging
VTI: velocity time integral
SV: stroke volume
HR: heart rate
CO: cardiac output
LVOT: left-ventricular outflow tract
e': mitral annular velocity during early diastole
sPAP: systolic pulmonary artery pressure
mPAP: mean pulmonary artery pressure
TVI: tissue velocity imaging
RV: right ventricular
FPRNA: first pass radionuclide angiocardiography
ERNA: equilibrium radionuclide angiocardiography
CMR: cardiac magnetic resonance imaging.
